# Impact of an integrated obesity management system on patient’s care - research protocol

**DOI:** 10.1186/s40608-014-0019-z

**Published:** 2014-09-03

**Authors:** Jean-Patrice Baillargeon, Denise St-Cyr-Tribble, Marianne Xhignesse, Andrew Grant, Christine Brown, Marie-France Langlois

**Affiliations:** 1 Division of Endocrinology, Department of Medicine, Université de Sherbrooke, Sherbrooke, Québec Canada; 2 École des sciences infirmières, Université de Sherbrooke, Sherbrooke, Québec Canada; 3 Department of Family and Emergency Medicine, Université de Sherbrooke, Sherbrooke, Québec Canada; 4 Department of Biochemistry, Université de Sherbrooke, Sherbrooke, Québec Canada

**Keywords:** Primary care, Obesity, Lifestyle, Medical education, Continuum of care

## Abstract

**Background:**

The majority of obese subjects are treated by primary care physicians (PCPs) who often feel uncomfortable with the management of obesity. In a previous study, we successfully developed, implemented and evaluated an obesity management system based on training and coaching of health professionals of family medicine groups (FMGs) by a team of experts in obesity management. Using a pre/post design, this study suggested a positive impact on health professionals’ perceptions and reported obesity care. The current research project is aimed at evaluating the impact on obesity screening and care of this integrated obesity management system. We hypothesize that our program combining preceptorships with a virtual community and on-site coaching will improve: (1) management and weight loss of obese/overweight subjects treated by PCPs for hypertension, type 2 diabetes or impaired glucose tolerance; and (2) screening and initial management of obesity among a regular follow-up group of patients of PCPs who practice in FMGs.

**Methods/Design:**

Ten FMGs will be approached for a practice monitoring project and will be randomised to receive the intervention developed in our previous project or will only be provided clinical practice guidelines. In the participating FMGs, we will enrol 22 patients per FMG with weight related targeted disease and 24 patients with regular follow-up. These patients will be evaluated for the care they received regarding screening and/or management of obesity using medical chart reviews, and will fill out a questionnaire on their lifestyle and satisfaction. They will also be examined for anthropometric measures, vital signs, blood markers for chronic diseases and physical fitness. The same patients will be assessed again after 18 months. The impact of the program on health professionals will be evaluated at baseline, and at 1 year. Qualitative data will also be collected from both professional and patient participants. Direct and indirect costs and QALYs will be evaluated as indicators of cost-effectiveness.

**Discussion:**

In the context of the dramatic increase in obesity prevalence and the low perception of PCPs’ self-efficacy, providing efficient strategies to PCPs and interdisciplinary health care teams for management of obesity is crucial.

**Trial registration:**

Clinicaltrials.gov Identifier: NCT00991640

## Background

### Obesity and management recommendations

Obesity is a major public health problem that was identified as an epidemic by the World Health Organization (WHO) and is associated with multiple co-morbidities, including type 2 diabetes (DM2), hypertension (HTN), cardiovascular disease and cancers [1]. As of 2004, nearly 60% of the Canadian population was reported as being overweight or obese [2]. Over the last 18 years, the proportion of the population considered to be obese has increased by 53% in Canada, but fortunately, the prevalence now seems to have stabilized [2]. Overweight and obesity affect all age groups, including approximately 1 out of 4 children and adolescents [2–4]. The direct cost of obesity in Canada was recently estimated at 4.3 billion dollars, in addition to 5.3 billion dollars related to sedentary lifestyles: these costs represent 4.8% of the global Canadian health care system’s budget [5]. Nonetheless, moderate weight loss in obese subjects has been proven to markedly ameliorate DM2, blood pressure, lipid profile and may decrease mortality [6–10].

In response to the alarming evolution of obesity in Canada, the 2005 federal budget provided $300 million over 5 years for a strategy focusing on healthy living and prevention of chronic disease [11]. Moreover, from 2006 to 2012, the Government of Quebec undertook an important action plan to promote healthy lifestyles and to prevent obesity related health problems. This program, called “Investing in the future”, consists of interventions aiming to: i) promote healthy nutrition, physically active lifestyles and favorable social norms; ii) improve services for overweight and obese individuals and; iii) encourage research and knowledge translation. Their goals for 2012 are to reduce by 2% the prevalence of obesity and that of overweight by 5%, among children and adults in Quebec [12]*.* Our research proposal is thus completely in line with these objectives and is developed in partnership with our local health agency (ASSSE) and the Quebec Ministry of Health (MSSS).

The “2006 Canadian Clinical Practice Guidelines on the Management and Prevention of Obesity in Adults and Children” (CCPGO) emphasize the importance of a multidisciplinary health care team for weight management and include evidence-based key recommendations for the clinical management of obesity [4]. They suggest a stepwise obesity and overweight management algorithm which can be applied in primary care. Furthermore, CCPGO put a special emphasis on the need for research to develop and evaluate the organization of care for overweight and obese individuals and also on the need for continuing education to focus on activities that provide physicians and health professionals with the skills to counsel people confidently. Our project is in line with these guidelines and will also help in appropriate knowledge transfer of the CCPGO that is needed in order to implement them in clinical practice.

### Inter-professional collaboration and organization of primary health care services

The implementation of family medicine groups (FMG) in 2001 resulted in major organizational changes in primary health care services in Quebec [13]. A FMG is a group of family physicians who practice together in collaboration with nurses and other health care professionals and are responsible for ensuring primary care services 24/7 in a given territory. Improved accessibility and continuity, interdisciplinarity and patient registration are central principles of that reorganization. FMGs provide further opportunities to improve care for specific conditions that warrant lifestyle modifications and call for interventions that are based on inter-professional collaboration. This construct refers to individuals from distinct disciplinary backgrounds, who work together to achieve a common goal. In today’s delivery of health care services where different professionals, especially physicians and nurses, are bound to work together, a collaborative approach may enhance quality of care and quality of life for patients [14–16]. A collaborative approach also involves patients and is reputed to promote their empowerment giving them the support to i) mobilize their resources; and ii) focus on their strengths allowing them to take charge of their situation [16]. Thus, obesity represents a special challenge that is increasingly addressed in some FMGs and benefits from a collaborative approach as outlined in the CCPGO [4,17].

### Models of care for chronic disease

The chronic care model (CCM) was developed and validated by the MacColl Institute for Healthcare Innovation and constitutes an organizational approach to caring for people with chronic disease [18,19]. It describes practical, supportive, evidence-based interactions between an informed, activated patient and a prepared, proactive practice team. The CCM identifies essential elements of a health care system that encourage high-quality chronic disease care including: the community; the health system; self-management support; delivery system design; decision support, and clinical information systems [18,20,21]. A meta-analysis of 112 studies has shown that interventions that contained one or more CCM elements improved clinical outcomes and processes of care [22]. This framework was used to develop our intervention.

It was complemented by a theoretical model for changing lifestyle behaviour developed by Maryon-Davis: the Three ‘*Es*’ Model which includes *Encouragement*, *Empowerment* and *Environment* [23]. Encouragement refers to support patient efforts to change their lifestyle mainly in regard to diet and physical activity. This support can be provided by allied health professionals, primary care physicians (PCPs) or even through media and social programs. Encouragement needs to be supported by empowerment, which is based on patient education and the acquisition of effective skills to concretely modify behaviour. The third E, which may be the most important, refers to the alliance of the cultural, social, physical and economic environments required to assist in lifestyle factor improvements such as nutrition and exercise. Interestingly, this model can also be applied to PCPs: increasing their knowledge, giving them accurate ways to manage obesity and offering them the support of an expert team as ways of increasing their self-confidence and self-efficacy which should ultimately have positive effects on their clinical practice and patients.

Thanks to inter-professional collaboration, expert-supported FMGs are an ideal setting for obesity management according to CCM and the Three “Es” Models as they can provide education, support and regular follow-up to patients within the health care system.

### Obesity management in primary care

Considering the growing prevalence of obesity, the majority of patients should be managed by primary care givers. Unfortunately, PCPs and health professionals often feel that they are unable to help their patients lose weight and their self-efficacy in obesity treatment is poor [24,25]. As a result, obesity tends to be neglected when compared to other chronic conditions like hypertension and diabetes. When auditing medical records from PCPs, obesity is definitely underreported and recommendations for weight control interventions are reported even less [26,27]. It stands to reason that if obesity is not mentioned in a patient’s record, it is unlikely that any intervention is ongoing. This only highlights the need for major changes with respect to medical practice regarding this important health problem.

Since 2004, a United Kingdom team set up a programme for weight management in primary care: the “Counterweight Programme” [26]. They recruited a total of 80 practices from seven different regions of the United Kingdom, of which 18 were randomly assigned to act as controls. In each practice of the intervention group, 50 obese and 50 age- and sex-matched normal weight patients were selected for a total of 1906 patients. At baseline, less than 58-71% of patients had a BMI measurement and only 13-16% of obesity diagnoses were recorded, supporting suboptimal screening and under-reporting of this condition in primary care. Primary care nurses (PCNs) were identified as the most suitable individuals to deliver the weight management intervention and received 12 h of training followed by 6 months of mentoring. The PCNs were encouraged to see patients previously referred by PCPs for six individual appointments or for six group sessions during a period of three months followed by quarterly meetings. Evaluation of the impact of the interventions on patients was then made at 12 and 24 months. Forty-nine percent of patients were considered as completers of the programme (based on follow-up compliance). At 12 months, 40% of completers and 33% of all patients followed-up (including non-completers) achieved a ≥5% weight loss that was mostly maintained at two years. This suggests that empowerment and education of patients in a primary care setting can have a successful impact on modification of their health behaviour and weight.

### Electronic tools in obesity management

Coupling a preceptorship with a virtual learning community (as further described below) and specific tools for the practice setting can be key ingredients to effect change in practice. Internet based communication, accessed at one’s own time in the clinic or at home, or used for same-time meetings, is transforming the continuing information environment. *e*Learning is becoming increasingly popular and has been proven to be at least as effective as classical continuing medical education (CME) to improve knowledge, and may initiate changes in medical practice [28].

This learning web-based architecture supports different dimensions and different theoretical considerations, including cognitive, educational, organizational, sociological, technical and other disciplines, all of which underlie the creation of this architecture. The various actors, specialists and community care givers, physicians, nurses, nutritionists and other team members, patients and their families make up a learning community. The term “virtual learning community” has recently gained common use where supported by web based communications [29–31]. It defines the cooperative process and is not restricted to structured courses. They follow integrative global objectives and require facilitation and technical expertise if they are to be sustained. Potential advantages of an *e*Learning community include a more fluid communication across the different actors enabling consensus or resolving of complex situations as well as flexibility with respect to different working environments and ethos [29–31].

### Contributions from our group and preliminary data

Our group has implemented an interdisciplinary approach to obesity care management since 2001. Our interdisciplinary team includes nurse-clinicians, dietitians, a psychologist, kinesiologists and several endocrinologists. It offers, under the coordination of the nurse-clinician, a variety of behavioural and motivational approaches to lifestyle modification through a series of group seminars in addition to individual consultations with health professionals every 6 weeks, or as needed. To our knowledge, such a system without out of pocket costs to the patient is almost unique in Canada. Prospective studies of obese patients with metabolic co-morbidities managed by our multidisciplinary team reveal that 45-55% lose 5% or more of their initial body weight after 12 months in our program [32,33]. This is accompanied by important metabolic benefits [33,34]. The effectiveness of our intervention is thus comparable to other effective but more intensive and costly interventions [8,9,35], making it more feasible in the context of the Canadian health care system.

**Figure 1:**
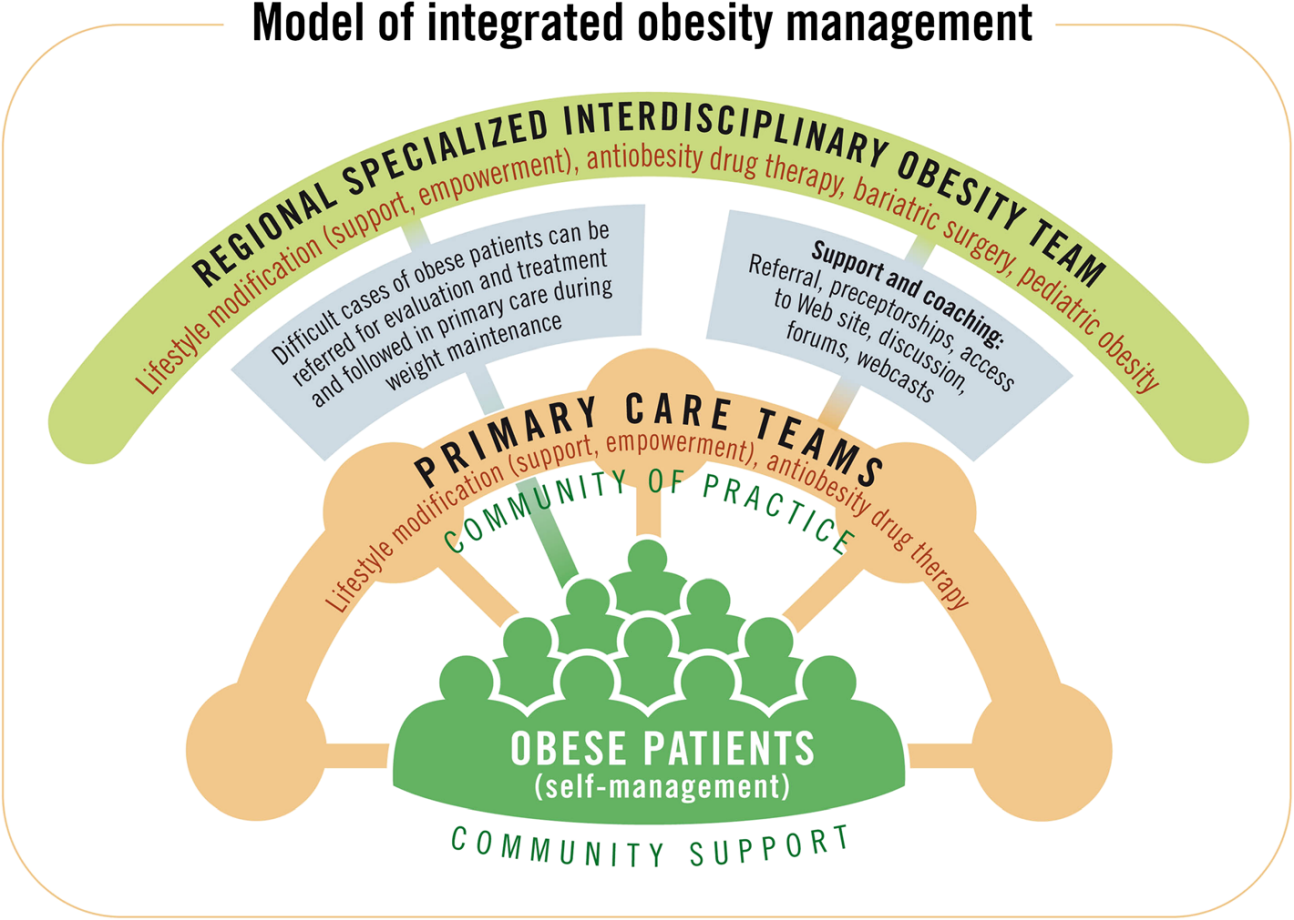
**Model of integrated obesity management.**

We have developed a model, based on the chronic care model, emphasizing the importance of primary care supported by regional experts, to improve obesity management throughout the continuum of care (Figure 1). In 2005, we obtained a first CIHR-PHSI grant to develop, implement and evaluate an obesity management system based on preceptorships in combination with a virtual community favouring continuous support of FMGs by our expert team [36]. This program aimed to enhance primary care teams’ expertise and attitudes with respect to obesity management, foster the implementation of nurse-coordinated team management for obesity in primary care settings, and improve access to quality support resources for PCPs and their teams, as well as their patients. Thirty-eight participants (13 nurses, 25 MDs) from 8 FMGs were enrolled in the project [37] and evaluated at baseline and 1 year later.

After an extensive needs assessment, a 2-day preceptorship was developed combining interactive sessions with experts, case discussions and observation of real patient encounters [38]. The preceptorship was validated and remodeled with the participation of one of the FMGs before being offered to all participants. A web-based portal with professional and patient tools in association with monthly interactive on-line educational activities for health professionals was implemented in tandem with preceptorships as the key ingredients used to effect change in practice [39].

Health professionals’ confidence level, attitude toward obese individuals and perception of self-efficacy to advise on exercise and diet improved after the preceptorships and these improvements were maintained 1 month and 1 year after as compared to baseline [25]. Clinical practice also seemed to improve with an increase in self-eported waist circumference measurement, evaluation of patient readiness to change and suggested use of a pedometer and food diary [25].

Our next step is now to evaluate the impact on health professionals’ clinical practice and patient-related outcomes of such an integrated model of obesity management and to move from a pre-post design to the stronger design of a randomized-controlled trial (Phase-2).

### Hypothesis

We hypothesize that our program combining preceptorships with a virtual community (intervention) will improve: (1) screening and initial management of obesity among unselected patients of PCPs who practice in FMGs; and (2) management and weight loss of obese and overweight subjects who are treated by PCPs for HTN, DM2 or impaired glucose tolerance (IGT). We further hypothesize that: (3) these benefits will be achieved through improvement of primary care providers’ confidence and competence in managing obese patients, as well as changes in their clinical practice of obesity care; (4) patients will express better satisfaction regarding their management; and (5) our research program will result in generalizable and transferable new knowledge useful for decision-makers of the healthcare system.

### Aims of the study

#### Primary aims

1. Among patients who are regularly followed by a PCP for weight-related diseases targeted for lifestyle management (HTN, DM2 or IGT) and with a BMI ≥ 25 (*targeted diseases group*), we aim at improving, after 18 months of participation in our program (vs. control):
  - the proportion of subjects who have an initial weight management intervention planned;
  - the proportion of subjects who lose at least 5% of their initial weight;
  - weight, waist circumference, lean body mass, blood pressure, physical fitness level, physical activity level and healthy eating habits;
  - biochemical markers of metabolic control and cardiac risk (HbA1c, ApoA and ApoB).
2. Among patients who are regularly followed-up by a PCP for other health conditions (*regular follow-up group*), we aim at improving after 18 months of participation in our program (vs. control):
  - the proportion of patients who have measured weight, BMI and waist circumference;
  - the proportion of overweight or obese subjects who have an initial intervention planned;
  - weight, waist circumference, lean body mass, blood pressure, physical fitness level, physical activity level, healthy eating habits and markers of metabolic control in overweight or obese subjects.

#### Secondary aims

3. Among health professionals who receive the intervention (vs. those who did not), we aim at:
  - ameliorating their attitudes and perceptions towards patients and treatment effectiveness;
  - improving their perception of self-efficacy in managing obesity;
  - increasing their knowledge and expertise on obesity management; and
  - changing their practice.
4. Among patients who are followed by PCPs (both targeted disease and regular follow-up groups) who receive the intervention (vs. control), we aim at:
  - evaluating their attitudes and perceptions regarding obesity/overweight and lifestyle; and
  - improving their satisfaction regarding their management.
5. Evaluate costs and indicators of cost-effectiveness of the intervention.
6. Transfer knowledge to intervening parties and decision-makers of the health system using embedded knowledge transfer to patients, health professionals and decision-makers in addition to traditional end-of-grant strategies.

## Methods/Design

### Research design

**Figure 2:**
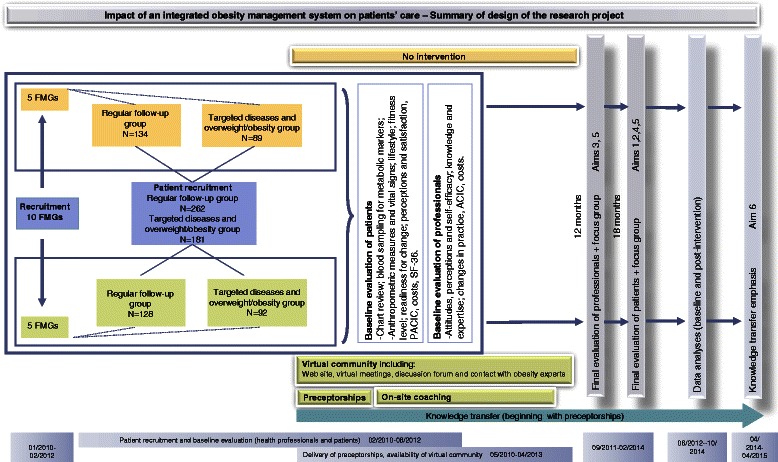
**Summary of design of the proposed research project.**

A schematic representation of research design is presented in Figure 2.

#### Study participants

The Integrated University Health Network, known as the RUIS (*Réseaux universitaires intégrés de santé*), of *Université de Sherbrooke* caters to a population of over 1 million people, encompassing a vast territory including Sherbrooke and Montérégie areas. A pool of at least 35 FMGs in this coverage area did not participate in our Phase-1 project. We will enroll a total of 10 of these FMGs in Phase-2, from which 4-10 members (including at least 2 PCPs and 1 nurse) will participate in our study. When we will initially contact FMGs, we will propose to them a study assessing lifestyle management in primary care and its evolution over time. When we will be in contact with 2 interested FMGs, we will randomize them to the intervention or control groups. The first FMG will then be offered to participate in our obesity management system with preceptorships and virtual learning community. The control FMG will be offered practice monitoring without being aware of the intervention. Participants of each group will sign different consent forms.

As much as possible, we plan to evaluate participants from FMGs pairs of intervention and control groups concomitantly in order to avoid bias related to time of evaluation (due to seasonal effects on weight, campaigns of health promotion, high-impact scientific publications, etc.). Within each FMG, adult patients who present for follow-up will be offered by FMG clerical personnel to have their chart accessed by our team and to be contacted if eligible for the project. If they consent, their chart will be screened to verify if they had, during the past 2 years, at least 4 documented visits for the targeted disease group and at least 2 visits for the regular follow-up group, and if continued follow-up is planned or highly probable in the next 18 months. We aim to include in our study 22 patients per FMG in the targeted disease group, and 24 patients per FMG in the regular follow-up group (total of 460 patients). Patient numbers will be prospectively accrued within each patient category during the recruitment period and before preceptorship delivery for the intervention group, until targeted numbers are achieved. In order to minimize important potential biases, patients will be kept blinded regarding their FMG group and the study objectives related to the evaluation of this intervention.

#### Intervention

The educative intervention is based on the format developed during our Phase-1 project, i.e. a two-day preceptorship combining theoretical and practical CME, and includes both plenary and nurse- or PCP-specific sessions. At the beginning of our Phase 2 project, we thoroughly reviewed the content of the preceptorship according to expert opinions relevant literature and comments/suggestions from Phase-1 participants. All important topics concerning CCPGO recommendations are covered, such as initial evaluation of patients (including readiness for change), nutritional approach, physical activity, behaviour modification and support to patients, pharmacotherapy, indications for bariatric surgery, obesity in children and adolescents etc. Training is divided into two days that are provided approximately 1 month apart in order for participants to put new knowledge into practice and better identify areas to emphasize during the 2^nd^ day. More than half of the time is devoted to the observation of real patient encounters and case discussions. Sessions are given by members of our research team who are also involved in the Centre Hospitalier Universitaire de Sherbrooke (CHUS) obesity clinic: endocrinologists, pediatric endocrinologist, dietician, kinesiologist and psychologist, according to their field of expertise. The general objective of the preceptorship is to increase self-efficacy and optimize interventions of health professionals regarding obese and overweight patients in complementarity with specialized interdisciplinary resources of the CHUS obesity clinic.

The intervention group also has access to the virtual community previously described, including the web site posting information for health professional and patient tools, monthly virtual meetings where advances in obesity management and difficult cases are discussed, and a discussion forum for participants and obesity experts.

Following expressed needs of participants at the end of Phase-1, we are also providing on-site coaching sessions for FMG participants every three months. They consist of 1 hour discussions with the dietician or kinesiologist of our team who visits the FMG to solve problems with web-site use, solve difficult cases, discuss use of program tools and explore practice changes. The objective is to help participants integrate the virtual community in their practice and answer their clinical questions.

The FMGs allocated to the control group are only provided with a copy of the current Canadian guidelines for the treatment of obesity, diabetes, dyslipidemia and HTN [4,40–42].

### Ethical considerations

The research project was reviewed and approved by the appropriate institutional Research Ethics Review Boards according to applicable laws and Tri-Council Policy Statement. Research participants (both patients and health professionals) were duly informed and consent was obtained in writing prior to participation. However, as mentioned above, subjects were not informed regarding the intervention. They were told that the objective of the study was to evaluate lifestyle management in primary care settings and its evolution over time. In order to minimize deceptive ethical issues, all participants will be fully informed of all the objectives and interventions of the study after their final assessment, with appropriate debriefing. Initial contact of potential candidate patients was performed by FMG clerical personnel who gave them a pamphlet with information on the study and obtained their signed consent to have their chart reviewed by the research team and to be contacted if needed. Ethical considerations raised by the research are mainly confidentiality issues. All data will be coded, archived for at least 5 years and then destroyed.

### Variables and research tools

#### Evaluation of patients

All evaluations will take place at baseline and after 18 months in order to allow sufficient time following the intervention to monitor changes in practice. Although very long term weight maintenance cannot be assessed during that period, we could eventually continue following patients on a longer term as part of future projects.

#### Anthropometric measures and vital signs

Weight will be measured in patient participants by a standard calibrated scale in kilograms to the nearest decimal (0.1 kg) wearing indoor clothing, with empty pockets and without shoes. Height will be measured with a stadiometer in meters to the nearest millimeter (0.001 m), without shoes. Waist circumference will be measured twice with a measuring tape at the top of the upper iliac crest, at the end of a normal expiration, as recommended; we will record the average of 2 measures with less than 1 cm variation [4,43]. Lean body mass, fat mass (in kg) and percent body fat will be measured by standing electric bioimpedance using a Tanita weight scale (model TBF-300A). Standing electric bioimpedance was shown to be reliable compared to underwater weighing and to conventional supine tetrapolar bioimpedance [44–46]. Blood pressure and pulse rate will be measured after five minutes of rest, in the sitting position. The average of two measurements will be used for analysis.

#### Medical history and lifestyle interventions

At the end of the study for each study site, thorough medical chart review will be performed using a standardized evaluation grid, also verifying medical and surgical history and medications for the 18 months before and after baseline evaluation of patients. Evaluations and interventions that are proposed to patients will be compared to that proposed in the CCPGO algorithm, and evolution of patients’ weight during the observation period will be recorded.

#### Metabolic markers

We chose metabolic markers not influenced by a fasting state in order to increase feasibility of blood sampling during patient evaluations. We will use HbA1c which correlates with glycemic control in patients with DM2 and is also a marker of dysglycemia which can be used to diagnose pre-diabetic states and DM [47]. Furthermore, HbA1c was identified as a good marker of cardio-vascular events even in non-diabetic individuals [48]. Lipoproteins ApoB (marker of atherogenic LDL particules) and ApoA1 (marker of protective HDL particules) will be used [42,49,50]. Apo B is thought to be a better marker of cardiovascular risk than LDL and a better predictor of treatment benefits [40,49,51]. Furthermore, ApoA1 and ApoB/ApoA1 ratio are expected to improve with weight loss and lifestyle modification and can be used as biomarkers of improved cardio-vascular risk [49,50,52,53]. These will be measured in the CHUS clinical biochemistry laboratory using standard methods.

#### Lifestyle

Physical activity level and eating habits will be measured using a questionnaire adapted from the one used by Statistics Canada for the latest Canadian Health Survey [54]. It inquires about the frequency and duration of active travelling, leisure time, and sedentary activities. We chose this questionnaire since it is easy and quick to complete and allows comparison of the characteristics of our patients to those of the general Canadian population.

#### Fitness level

Since objective measurements of exercise capacity in patients are usually better than self-reports, we will use the six-minute walk test (6MWT), a simple index that estimates functional capacity in obese subjects and is also a predictor of morbidity and mortality [55–57]. The 6MWT is a simple test that we will be performed in FMGs where the environment permits according to the protocol recommended by the American Thoracic Society, and measures the distance that a patient can quickly walk in a period of 6 minutes [58]. It has been shown that weight loss increases functional capacity, as measured by the 6MWT, early in the weight loss process, making this test a very accurate tool to evaluate the impact of the intervention on obese/overweight patients’ functional capacity [59–61].

#### Readiness for change

In the course of previous prospective studies, we have designed a 22-question weight loss readiness tool (WLRT) based on Prochaska and DiClemente’s Stages of Change Model, which is currently being validated [4,62]. It evaluates the motivational readiness of patients regarding weight management, nutrition and physical activity, and could predict response to an intervention. Our WLRT takes 5-10 minutes to fill out, making it a clinically applicable tool. It has been useful to identify subjects with greater chances of success for lifestyle modification in previous studies of our group [63,64]. Its predictive value will be assessed in this study, but it will also serve to explore the mechanisms of potential benefits of our intervention and for stratified analyses.

#### Conformity to CCM

We will be using the Patient Assessment of Chronic Illness Care questionnaire (PACIC), developed and validated by the McColl Institute to evaluate perceived concordance of received care with the CCM [18,20,21]. We used the World Health Organization process of translation and adaptation of instruments to translate the questionnaire into French [65].

#### Perceptions and satisfaction of patients

Patient perceptions of their personal experience regarding weight management and their satisfaction regarding management by their health professionals will be evaluated with questionnaires that we designed based on previous studies. A qualitative in-depth analysis of patients’ perceptions will also enhance our understanding of their personal experience with the care received for lifestyle optimization in FMGs. Eight to ten patient volunteers will be recruited in each FMG to participate in a taped-recorded semi-structured qualitative interview to assess their perceptions of impact of the program (continuity of care, perceptions of changes, contribution to change, quality of life, perspectives of future changes, explanation of level of satisfaction with the program). An interview guide with open-ended questions derived/adapted from the principal variables of the study, the Diabetic Empowerment Scale (DES) [16], and the Weight-related Quality of Life (WQOL-lite) questionnaire [66] will be used as in previous studies from members of our group [33,67].

#### Evaluation of health professionals (PCPs and nurses)

All evaluations will take place at baseline, T1 (1 month after the preceptorship for the intervention group and 1 month after baseline for the control group) and 12 months after T1 in order to compare the data collected during our Phase 1 study and to compare intervention and control groups. The same questionnaires that we developed and validated will be used to evaluate Aim 3 of this proposal. We have slightly modified some tools based on the results of our Phase 1 project. Results will be correlated to patients’ outcome.

#### Attitudes, perceptions and self-efficacy toward obesity management

Self-efficacy is defined as the set of beliefs about one’s capabilities to perform at a designated level [68]. For health professionals to engage in weight management with their patients, they must not only accept this as part of their role but also feel that they are competent to accomplish the task. Indeed, 3 stages were identified in physician learning [69]: i) deciding whether to take on a learning task to address a problem; ii) learning the skill and knowledge anticipated to resolve the problem and iii) gaining experience in using what has been learned. Negative attitudes toward obese individuals and perceptions to the effect that available treatments are ineffective (as found at baseline in our Phase-1 study), can prevent the engagement of health professionals in weight management [24,25,70]. Thus, by providing participants with the necessary knowledge, skills and continued support to undertake weight management with their patients, we believe that we will be able to initiate what Tiberius and Tipping considered a “healthy spiral”: beginning with an acceptance of inherent challenges (related to weight management) and moving to success at those tasks (helping patients) and finally to the development (through experience) of a more positive sense of self-efficacy [71]. Changes in attitudes and perceptions could be correlated to changes in practice and patient outcomes. This will be evaluated using questionnaires that we developed in our previous project based on the literature.

#### Knowledge and expertise on obesity management

To assess the impact of our intervention on medical knowledge regarding obesity, physicians and nurses will be asked to respond to a short-answer and multiple-choice questionnaire of 25 questions. The questionnaire will address topics related to obesity management such as physical activity and nutritional recommendations a and non-obesity related topics such as management of hypertension or tobacco cessation.

#### Conformity with CCM

We will be using the Assessment of Chronic Illness Care questionnaire (ACIC version 3.5), developed and validated by the McColl Institute to evaluate the organization of care in the FMG corresponding to each element of the CCM [18,20,21,72].

#### Changes in practice

A services evaluation grid will be completed with the administrator of the FMG in order to assess practice organization. Also, individual practice of participants will be assessed using clinical vignettes describing a patient presenting for a routine exam or for weight management. This tool was used in our previous study to demonstrate changes in reported management of obesity. A qualitative in-depth analysis of participants perceptions of the program will be performed using semi-structured interviews in each FMG [36]. This will increase our understanding of their experience, inter-professional collaboration, satisfaction with the program, change process and further identify and detail strengths and areas for potential improvement.

#### Evaluation of cost-effectiveness

We will thoroughly evaluate direct and indirect costs of implementation and delivery of our intervention, which are multiple: costs for the instigators of the program (using a log book for all team members involved in delivery of the intervention, etc.); costs for FMG professionals; costs for visits to other health care facilities where patients seek care for their specific problem of obesity/overweight; costs for patients; and costs to society modelized in a Markov model using data from the literature and the study [73,74]. Once data on all of those costs will be collected and compared between the two groups, a sensitivity analysis will be conducted with different scenarios over several years and with different discount rates to reflect the uncertainty of some variables [75].

#### *Quality Adjusted Life Years (QALYs*)

It is now recognized that to better treat the patient, and not only the disease, doctors should take into account the patient’s quality of life related to health. We will thus be using the most used questionnaire, the SF-36, derived from the “Medical Outcome Study” completed by patient-participants [76]. To address the most important limitations of the SF-36 and build an instrument that allows us to conduct a cost-effectiveness analysis, we will apply the transformation of Brazier et al. [77] to obtain QALYs based on stated preferences of individuals through the method of “Standard Gamble” (lottery). This transformation allows to take into account: 1) the existence of differences in individuals’ preferences between different dimensions of health, and 2) the possibility that the interval between different possible answers to each question in the SF-36 is not equal. The transformation model chosen will be model number 10 [78]. A complementary measure of QALYs will also be used to ensure consistency and continuity of results. This additional measure is to apply a time trade-off issue in the preferences of individuals [79]. This issue will be formulated as follows: “We make the assumption that for reasons that cannot be changed, you only have 10 years to live. Of these 10 remaining years to live, how many years of life would you be willing to give up to live the remaining years with a weight 20% less than what your weight is now?”

### Sample size, data analysis and interpretation

According to AIM 1, our primary variable of interest is the proportion of obese or overweight subjects with HTN, DM2 or IGT who will lose least 5% of their initial weight after eighteen months. Assuming that 30% drop-out or provide incomplete data, our study needs to recruit 220 obese or overweight subjects with HTN, DM2 or IGT in order to achieve 80% power (using chi-square) to detect a doubling in the proportion of those who will lose at least 5% of their initial weight, based on a proportion of 21% in the control group (which is the average proportion observed in the control groups of two of our previous studies) [32,33,67] and 42% in the intervention group (which represent half of the average benefits found in these two studies compared to control groups) (***AIM 1***). Similarly, 240 unselected patients provided 80% power (using Fisher’s-exact test because of small minimum expected cell size) to detect an improvement of the proportion of these patients who have a reported measure of BMI from 80% in the control group (based on reported practice in our previous participants) to 95% in the intervention group (which is close to the 100% recommended by the CCPGO) (***AIM 2***). For AIM 2, our primary variable of interest is the proportion of unselected patients who have a reported measure of BMI, which defines obesity according to WHO and is therefore essential for the screening of obesity.

The impact of our program on patient’s care will be assessed by the comparison of patients’ outcome variables between those recruited from FMGs allocated to intervention and those recruited from FMGs allocated to no intervention. Similarly, the impact of our program on PCPs will be determined by the comparison of variables related to PCPs between those who received the intervention and those who did not. Univariate analyses will use two-tailed unpaired *t* tests to compare continuous variables and chi-square or Fisher’s-exact tests to compare categorical variables. Continuous variables that are not normally distributed will be log-transformed in order to ascertain normal distribution, whenever possible, or will be compared using two-tailed Wilcoxon tests. Multivariate analyses will also be used in order to adjust for baseline differences between groups, potential confounders and cluster effects of individual FMGs. Should differences between patients’ or PCPs’ groups be evidenced, Pearson correlation analyses will be performed to determine if such differences are related to a potentially mechanistic variable. For example, benefits in patients’ outcome variables will be correlated with changes in variables assessing attitude, perceptions, knowledge and/or better clinical practice for their primary care providers. An α level of 5% will be used for all analyses.

### Knowledge translation plan *(Aim 5)*

It is worth mentioning that our research program is optimal to allow knowledge translation (KT) and application throughout the entire research process. The preceptorship experience in itself is a knowledge translation activity for health professionals. Our group is also actively involved in developing a National Preceptorship program with the Canadian Obesity Network (CON) and this could evolve in the development of a national virtual community and improvements in CON CME activities. Evidence-based information on patient outcomes generated by our study will have a great impact on knowledge transfer: if beneficial, it would accelerate the implementation of such programs as practice models; and if benefits are less than expected, it will orientate to program modification.

However, research by itself does little to induce change (except for participants) and thus, results have to be diffused to interested audiences. We have access to specialized communication resources from the CHUS Research Center and CON to develop an optimal strategy for dissemination of results and potential applications.

Our research is relevant to PCPs, nurse coordinators, specialists in various disciplines and associated health professionals, CME departments, public health directorates, health-system decision-makers. We will reach out to these parties through linkage and exchange activities, including presentation of study results at scientific meetings and publication in scientific journals, but also by direct reports to decision-makers and health policy stakeholders. A clear summary of research results, including key messages targeted for each selected audience and synthesized results (divided by themes) will be available (printed and on our web site). Local and national media will be invited to press conferences and we will schedule private meetings with important decision-makers to whom our findings are relevant.

## Discussion

### Impact

The outcome measures of our proposal are relevant and useful to a number of health systems’ managers and policy makers. The ASSSE, as well as stakeholders from the Ministry of Health of Quebec, Canadian Obesity Network and FMGs, are decision makers active in this research project and results will impact on the planning, allocation and management decision of policy makers as they apply to service organizations. Early involvement of policy makers as co-investigators and active participants in our research project and their interactions with our team since 2005 will considerably increase the likelihood that they feel ownership of the findings. Their implication in our first partnership program was greatly appreciated and their interest is growing: they regard as very pertinent the assessment of direct impact on patients’ care as proposed in this project and positive results will greatly impact on their willingness to implement such interventions. Research results are anticipated to be applicable to other institutions and regions of Quebec and Canada because most regions have access to specialists who could support networking with a PCP team. Our evaluation will also allow us to improve the program and knowledge transfer will be greatly facilitated by our partnership with CON.

### Importance and generalizability

This project is very important as it generates new knowledge on cost-effective and applicable measures for the management of obesity and should improve access to care for obese and overweight patients by engaging primary health care teams. We know that obesity is an important public health problem that leads to adverse medical complications and that it can be treated effectively to reduce not only weight but also co-morbidities and mortality. However, comprehensive obesity management is seldom undertaken in primary care due to inadequate training, insufficient resources and poor self-efficacy. Our preliminary data show that the obesity management system developed by our team significantly improves perceptions, attitudes and low perceived self-efficacy of PCPs and PCNs and changes their reported practice. This is extremely promising, but evaluation of the impact of such a system on measured patient outcomes through the current project is necessary before changing a health care system’s organization. Generalizability of our findings is high since expert teams in the management of obesity are present throughout Canada (already networking through CON), and primary care is developing teams of PCPs and nurses in many Provinces. Also, this model could apply to the management of other chronic diseases and be adapted to other health care systems.

## Abbreviations

6MWT: Six-minute walk test
ACIC: Assessment of Chronic Illness Care questionnaire
ASSSE: Local health agency (*Agence de santé et services sociaux de l’Estrie*)
BMI: Body mass index
CCM: Chronic care model
CCPGO: Canadian Clinical Practice Guidelines on the Management and Prevention of Obesity in Adults and Children
CHUS: Centre Hospitalier Universitaire de Sherbrooke
CIHR: Canadian Institutes of Health Research
CME: Continuing medical education
CON: Canadian Obesity Network
DES: Diabetic Empowerment Scale
DM2: Type 2 diabetes
FMG: Family Medicine Group (*Groupe de Médecine de Famille)*
HTN: Hypertension
IGT: Impaired glucose tolerance
KT: Knowledge translation
MSSS: Ministry of Health of Québec
PACIC: Patient Assessment of Chronic Illness Care questionnaire
PCN: Primary care nurses
PCP: Primary care physician
PHSI: Partnerships for Health Systems Improvement
QALYs: Quality Adjusted Life Years
RUIS: Integrated University Health Network (*Réseaux universitaires intégrés de santé*)
WHO: World Health Organization
WLRT: Weight loss readiness tool
WQOL-lite: Weight-related Quality of Life questionnaire

## References

[1] WHO (1997). Obesity: preventing and managing the global epidemic. Report of a WHO consultation on obesity.

[2] Tjepkema M (2005). Measured obesity: adult obesity in Canada - measured height and weight. Nutrition: findings from the Canadian Community Health Survey 2004.

[3] Tremblay MS, Willms JD (2000). Secular trends in the body mass index of canadian children. CMAJ.

[4] Lau DC, Douketis JD, Morrison KM, Hramiak IM, Sharma AM, Ur E (2007). 2006 Canadian clinical practice guidelines on the management and prevention of obesity in adults and children [summary]. Cmaj.

[5] Katzmarzyk PT (2004). The economic cost associated with physical inactivity and obesity in Canada: an update. Can J Appl Physiol.

[6] Reisin E, Abel R, Modan M, Silverberg DS, Eliahou HE, Modan B (1978). Effect of weight loss without salt restriction on the reduction of blood pressure in overweight hypertensive patients. N Engl J Med.

[7] Williamson DF, Thompson TJ, Thun M, Flanders D, Pamuk E, Byers T (2000). Intentional weight loss and mortality among overweight individuals with diabetes. Diabetes Care.

[8] Tuomilehto J, Lindstrom J, Eriksson JG, Valle TT, Hamalainen H, Ilanne-Parikka P, Keinanen-Kiukaanniemi S, Laakso M, Louheranta A, Rastas M, Salminen V, Uusitupa M (2001). Prevention of type 2 diabetes mellitus by changes in lifestyle among subjects with impaired glucose tolerance. N Engl J Med.

[9] Knowler WC, Barrett-Connor E, Fowler SE, Hamman RF, Lachin JM, Walker EA, Nathan DM (2002). Reduction in the incidence of type 2 diabetes with lifestyle intervention or metformin. N Engl J Med.

[10] Lindstrom J, Ilanne-Parikka P, Peltonen M, Aunola S, Eriksson JG, Hemio K, Hamalainen H, Harkonen P, Keinanen-Kiukaanniemi S, Laakso M, Louheranta A, Mannelin M, Paturi M, Sundvall J, Valle TT, Uusitupa M, Tuomilehto J (2006). Sustained reduction in the incidence of type 2 diabetes by lifestyle intervention: follow-up of the Finnish Diabetes Prevention Study. Lancet.

[11] Health Canada: **The Government of Canada reaffirms its commitment to combat Canada’s rising obesity levels.** *ᅟ* 2005. Online http://www.collectionscanada.gc.ca/webarchives/20061212073922/ http://www.hc-sc.gc.ca/ahc-asc/media/nrcp/2005/2005_74_e.html.

[12] Service de Santé et des Services sociaux du Québec (2006). Investir pour l'avenir, Plan d'action gouvernemental de promotion de saines habitudes de vie et de prévention des problèmes reliés au poids 2006-2012. Québec: Gouvernement du Québec.

[13] Clair M, Aucoin L, Begman H, Côté R, Ippersiel P, LeBoutillier J, Limoges GA, Rajotte H, Trépanier V, Rouleau R (2000). Commission d'étude sur les services de santé et les services sociaux. Les solutions émergentes. Québec: Gouvernement du Québec.

[14] Henneman EA, Lee JL, Cohen JI (1995). Collaboration: a concept analysis. J Adv Nurs.

[15] D’Amour D (2002). La collaboration professionnelle: un choix obligé.

[16] St-Cyr-Tribble D, Gallagher F, Vanasse A, Doré C, Archambault J, Fortin M (2007). Programme d’intervention fondé sur un modèle de collaboration interprofessionnelle et de promotion de l’autosoin auprès des diabétiques de type 2. Rapport de recherche, FCRSS & Aventis Canada.

[17] Beaulieu MD, Denis JL, D'Amour D, Goudreau J, Haggerty J, Hudon É, Jobin G, Lamothe L, Gilbert F, Guay H, Cyr G, Lebeau R (2006). L'implantation des Groupes de Médecine de Famille: le défi de la réorganisation de la pratique et de la collaboration interprofessionnelle. Montréal: Chaire Docteur Sadok Besrour en médecine familiale; avril.

[18] Wagner EH, Austin BT, Davis C, Hindmarsh M, Schaefer J, Bonomi A (2001). Improving chronic illness care: translating evidence into action. Health Aff (Millwood).

[19] Barr VJ, Robinson S, Marin-Link B, Underhill L, Dotts A, Ravensdale D, Salivaras S (2003). The expanded chronic care model: an integration of concepts and strategies from population health promotion and the chronic care model. Hosp Q.

[20] Wagner EH (1998). Chronic disease management: what will it take to improve care for chronic illness?. Eff Clin Pract.

[21] Wagner EH, Glasgow RE, Davis C, Bonomi AE, Provost L, McCulloch D, Carver P, Sixta C (2001). Quality improvement in chronic illness care: a collaborative approach. Jt Comm J Qual Improv.

[22] Tsai AC, Morton SC, Mangione CM, Keeler EB (2005). A meta-analysis of interventions to improve care for chronic illnesses. Am J Manag Care.

[23] Maryon-Davis A (2005). Weight management in primary care: how can it be made more effective?. Proc Nutr Soc.

[24] Foster GD, Wadden TA, Makris AP, Davidson D, Sanderson RS, Allison DB, Kessler A (2003). Primary care physicians’ attitudes about obesity and its treatment. Obes Res.

[25] Baillargeon JP, St-Cyr-Tribble D, Xhignesse M, Grant A, Carpentier A, Donovan D, Fortin M, Simoneau-Roy J, Brown C, Champoux A, Langlois MF (2008). Obesity preceptorship and virtual community result in changes of primary care practice. 2008 Annual scientific meeting NAASO, Phoenix AZ, 3-7 Octobre 2008. #363-P (Poster). Obesity.

[26] McQuigg M, Brown J, Broom J, Laws RA, Reckless JP, Noble PA, Kumar S, McCombie EL, Lean ME, Lyons GF, Frost GS, Quinn MF, Barth JH, Haynes SM, Finer N, Ross HM, Hole DJ (2005). Empowering primary care to tackle the obesity epidemic: the Counterweight Programme. Eur J Clin Nutr.

[27] Bramlage P, Wittchen HU, Pittrow D, Kirch W, Krause P, Lehnert H, Unger T, Hofler M, Kupper B, Dahm S, Bohler S, Sharma AM (2004). Recognition and management of overweight and obesity in primary care in Germany. Int J Obes Relat Metab Disord.

[28] Wutoh R, Boren SA, Balas EA (2004). eLearning: a review of Internet-based continuing medical education. J Contin Educ Health Prof.

[29] Brown JS, Duguid P (1997). Organizational learning and communities-of-practice: toward a unified view of working, learning and innovation. Organ Sci.

[30] Von Krogh G (2002). The communal resource and information systems. J Strat Inform Syst.

[31] Pan SL, Leidner DE (2003). Bridging communities of practice with information technology in pursuit of global knowledge sharing. J Strat Inform Syst.

[32] Kamga-Ngande CN, Carpentier AC, Nadeau-Marcotte F, Ardilouze JL, Baillargeon JP, Bellabarba D, Houde G, Langlois MF (2009). Effectiveness of a multidisciplinary program for management of obesity: the Unite d’Enseignement, de Traitement et de Recherche sur l’Obesite (UETRO) database study. Metab Syndr Relat Disord.

[33] Gagnon C, Brown C, Couture C, Kamga-Ngande CN, Hivert MF, Baillargeon JP, Carpentier AC, Langlois MF (2011). A cost-effective moderate-intensity interdisciplinary weight-management programme for individuals with prediabetes. Diabetes Metab.

[34] Couture C, Gagnon C, Brown C, Kamga CN, Hivert MF, Baillargeon JP, Langlois MF, Carpentier A (2007). Weight loss is associated with improvement in beta-cell function in subjects with impaired glucose tolerance. Obesity.

[35] Wadden TA, West DS, Neiberg RH, Wing RR, Ryan DH, Johnson KC, Foreyt JP, Hill JO, Trence DL, Vitolins MZ, Look ARG, Wadden TA, West DS, Neiberg RH, Wing RR, Ryan DH, Johnson KC, Foreyt JP, Hill JO, Trence DL, Vitolins MZ (2009). One-year weight losses in the Look AHEAD study: factors associated with success. Obesity.

[36] Baillargeon JP, Carpentier A, Donovan D, Fortin M, Grant A, Simoneau-Roy J, St-Cyr-Tribble D, Xhignesse M, Langlois MF (2007). Integrated obesity care management system -implementation and research protocol. BMC Health Serv Res.

[37] Baillargeon JP, Xhignesse M, Carpentier A, Donovan D, Grant A, Fortin M, St-Cyr-Tribble D, Simoneau-Roy J, Brown C, Champoux A, Langlois MF (2007). Programme intégré de prise en charge de l’obésité: recrutement et faisabilité. Mont-Tremblant, Qc: 4e Forum international francophone de pédagogie médicale.

[38] Xhignesse M, Baillargeon JP, Brown C, Champoux A, Langlois MF (2007). Évaluation des besoins dans le cadre du développement d'un préceptorat clinique en obésité. Mont-Tremblant, Qc*.* 4e Forum international francophone de pédagogie médicale.

[39] Caron P, Beaudoin G, Leblanc F, Grant A (2007). Architecture for implementation of a lifelong online learning environment (LOLE). Int Jl on E-Learn.

[40] Canadian Diabetes Association Clinical Practice Guidelines Expert Committee (2008). Canadian Diabetes Association 2008 clinical practice guidelines for the prevention and management of diabetes in Canada. Can J Diab.

[41] Canadian Hypertension Education Program (2011). 2011 CHEP Recommendations on management of hypertension. Ontario, Canada: Hypertension Canada.

[42] Genest J, McPherson R, Frohlish J, Anderson T, Campbell N, Carpentier A, Couture P, Dufour R, Fodor G, Francis GA, Grover S, Gupta M, Hegele RA, Lau DC, Leiter L, Lewis GF, Lonn E, Mancini GB, Ng D, Pearson GJ, Sniderman A, Stone JA, Ur E (2009). Canadian Cardiovascular Society/Canadian guidelines for the diagnosis and treatment of dyslipidemia and prevention of cardiovascular disease in the adult – 2009 recommendations. Can J Cardiol.

[43] Poirier P, Despres JP (2003). Waist circumference, visceral obesity, and cardiovascular risk. J Cardiopulm Rehabil.

[44] Cable A, Nieman DC, Austin M, Hogen E, Utter AC (2001). Validity of leg-to-leg bioelectrical impedance measurement in males. J Sports Med Phys Fitness.

[45] Spencer CE, Lingard JM, Bermingham MA (2003). Comparison of a footpad analyser with a tetrapolar model for the determination of percent body fat in young men. J Sci Med Sport.

[46] Gagnon C, Menard J, Bourbonnais A, Ardilouze JL, Baillargeon JP, Carpentier A, Langlois MF (2010). Foot-to-foot versus hand-to-foot bioelectrical impedance methods in a population with a wide range of body mass indices. Metab Syndr Relat Disord.

[47] American Diabetes Association (2010). Diagnosis and classification of diabetes mellitus. Diabetes Care.

[48] Santos-Oliveira R, Purdy C, Pereira Da Silva M, Dos Anjos Carneiro-Leao A, Machado M, Einarson T (2011). Haeniglobin A1c levels and subsequent cardiovascular disease in persons without diabetes: a meta-anlalysis of prospective cohorts. Diabetologia.

[49] Chan D, Watts G (2006). Apolipoproteins as markers and managers of coronary risk. Q J Med.

[50] Walldius G, Jungner I (2006). The apoB/apoA-I ratio: a strong, new risk factor for cardiovascular disease and a target for lipid-lowering therapy – a review of the evidence. J Intern Med.

[51] Barter PJ, Ballantyne CM, Carmena R, Cabezas MC, Chapman MJ, Couture P, De Graaf J, Durrington PN, Faergeman O, Frohlich J, Furberg CD, Gagne C, Haffner SM, Humphries SE, Jungner I, Krauss RM, Kwiterovich P, Marcovina S, Packard CJ, Pearson TA, Reddy KS, Rosenson R, Sarrafzadegan N, Sniderman AD, Stalenhoef AF, Stein E, Talmud PJ, Tonkin AM, Walldius G, Williams KMS (2006). Apo B versus cholesterol in estimating cardiovascular risk and in guiding therapy: report of the thirty-person/ten-country panel. J Intern Med.

[52] Behre C, Bergstrom G, Schmidt C (2012). Moderate physical activity is associated with lower ApoB/ApoA-I ratios independently of other risk factors in healthy, middle-aged men. Angiology.

[53] Gardner C, Tribble D, Young D, Ahn D, Fortmann S (2000). Associations of HDL, HDL2, and HDL3 cholesterol ans apolipoproteins A-I and B with lifestyle factors in healthy women and men: the Stanford Five City Project. Prev Med.

[54] ᅟCanadian Community Health Survey - Annual Component (CCHS). http://www23.statcan.gc.ca/imdb/p2SV.pl?Function=getSurvey&SDDS=3226.

[55] Hulens M, Vansant G, Claessens AL, Lysens R, Muls E (2003). Predictors of 6-minute walk test results in lean, obese and morbidly obese women. Scand J Med Sci Sports.

[56] Hulens M, Vansant G, Lysens R, Claessens AL, Muls E (2001). Exercise capacity in lean versus obese women. Scand J Med Sci Sports.

[57] Solway S, Brooks D, Lacasse Y, Thomas S (2001). A qualitative systematic overview of the measurement properties of functional walk tests used in the cardiorespiratory domain. Chest.

[58] American Thoracic Society (2002). ATS statement: guidelines for the six-minute walk test. Am J Respir Crit Care Med.

[59] Maniscalco M, Zedda A, Giardiello C, Faraone S, Cerbone MR, Cristiano S, Sofia M (2006). Effect of bariatric surgery on the six-minute walk test in severe uncomplicated obesity. Obes Surg.

[60] Lemoine S, Rossell N, Drapeau V, Poulain M, Garnier S, Sanguignol F, Mauriege P (2007). Effect of weight reduction on quality of life and eating behaviors in obese women. Menopause.

[61] Beriault K, Carpentier AC, Gagnon C, Menard J, Baillargeon JP, Ardilouze JL, Langlois MF (2009). Reproducibility of the 6-minute walk test in obese adults. Int J Sports Med.

[62] Norcross JC, Prochaska JO (2002). Using the stages of change. Harv Ment Health Lett.

[63] Kong W, Langlois MF, Kamga-Ngandé C, Gagnon C, Brown C, Baillargeon JP (2010). Predictors of success to weight-loss intervention program in individuals at high risk for type 2 diabetes. Diabetes Res Clin Pract.

[64] Domingue M, Baillargeon J, Brown C, Lebrun V, Langlois M (2011). Conviction and confidence for dietary changes predict early weight loss in a lifestyle modification intervention. Can J Diabetes.

[65] **Process of translation and adaptation of instruments.** http://www.who.int/substance_abuse/research_tools/translation/en/.

[66] Kolotkin RL, Head S, Hamilton M, Tse CK (1995). Assessing impact of weight on quality of life. Obes Res.

[67] Kamga CN, Carpentier A, Baillargeon JP, Dionne I, Ardilouze JL, Langlois MF (2005). Treatment of the Metabolic Syndrome in an interdisciplinary obesity clinic: a randomized controlled study. North American Association for the Study of Obesity’s 2005 Annual Scientific Meeting. Obes Res.

[68] Bandura A, Ramachaudran VS (1994). Self-efficacy. Encyclopedia of Human Behavior Orlando*.* edn.

[69] Geertsma RH, Parker RC, Whitbourne SK (1982). How physicians view the process of change in their practice behavior. J Med Educ.

[70] Wadden TA, Volger S, Sarwer DB, Vetter ML, Tsai AG, Berkowitz RI, Kumanyiaka S, Schmitz KH, Diewald LK, Barg R, Chittams J, Moore RH (2011). A two-year randomized trial of obesity treatment in primary care practice. New Engl J Med.

[71] Tiberius RG, Tipping J, Bess JL (2000). The discussion leader: fostering student learning in groups. Teaching alone teaching together: transforming the structure of teams for teaching*.* edn.

[72] Bonomi AE, Wagner EH, Glasgow RE, VonKorff M, Bonomi AE, Wagner EH, Glasgow RE, VonKorff M (2002). Assessment of chronic illness care (ACIC): a practical tool to measure quality improvement. Health Serv Res.

[73] Oster G, Thompson D, Edelsberg G, Bird A, Colditz G (1999). Lifetime health and economic benefits of weight loss among obese persons. Am J Public Health.

[74] Peeters A, Barendregt JJ, Willekens F, Mackenbach JP, Al Mamun A, Bonneux L (2003). Obesity in adulthood and its consequences for life expectancy: a life-table analysis. Ann Intern Med.

[75] Drummond F (2005). Methods for the Economic Evaluation of Health Care Programmes.

[76] Tarlov A, Ware J, Greenfield S, Nelson E, Perrin E, Zubkoff M (1989). The medical outcome study: application of methods for monitoring the results of medical care. JAMA.

[77] Brazier J, Roberts J, Deverill M (2002). The estimation of a preference-based measure of health from the SF-36. J Health Econ.

[78] Pickard A, Wang Z, Walton S, Lee T (2005). Are decisions using cost-utility analyses robust to choice of SF-36/SF-12 preference-based algorithm?. Health Qual Life Outcomes.

[79] Brazier J, Ratcliffe J, Tsuchiya A, Solomon J (2007). Measuring and Valuing Health and Economic Evaluation.

