# Erratum to: Impact of an integrated obesity management system on patient’s care - research protocol

**DOI:** 10.1186/s40608-016-0101-9

**Published:** 2016-04-29

**Authors:** Jean-Patrice Baillargeon, Denise St-Cyr-Tribble, Marianne Xhignesse, Andrew Grant, Christine Brown, Thomas G. Poder, Marie-France Langlois

**Affiliations:** Division of Endocrinology, Department of Medicine, Université de Sherbrooke, Sherbrooke, Québec Canada; École des sciences infirmières, Université de Sherbrooke, Sherbrooke, Québec Canada; Department of Family and Emergency Medicine, Université de Sherbrooke, Sherbrooke, Québec Canada; Department of Biochemistry, Université de Sherbrooke, Sherbrooke, Québec Canada; CRCHUS and UETMIS, CIUSSS de l’Estrie – CHUS, Sherbrooke, Québec Canada

After publication of the original article [1] the authors requested that the author list be updated. Therefore, we would like to acknowledge Thomas G. Poder as an author of this manuscript. The order of the author list has also been changed. The original order appeared as follows:

Jean-Patrice Baillargeon^1^, Denise St-Cyr-Tribble^2†^, Marianne Xhignesse^3†^, Andrew Grant^4†^, Christine Brown^1†^ and Marie-France Langlois^1*^

The new order, however, should be as listed below:

Jean-Patrice Baillargeon^1^, Denise St-Cyr-Tribble^2†^, Marianne Xhignesse^3†^, Andrew Grant^4†^, Christine Brown^1†^, Thomas G. Poder^5^ and Marie-France Langlois^1*^
